# Cytoplasmic genome types of European potatoes and their effects on complex agronomic traits

**DOI:** 10.1186/s12870-015-0545-y

**Published:** 2015-06-26

**Authors:** Rena Sanetomo, Christiane Gebhardt

**Affiliations:** Obihiro University of Agriculture and Veterinary Medicine, Potato Germplasm Enhancement Laboratory, West 2-11, Inada, Obihiro, Hokkaido 080-8555 Japan; Max-Planck Institute for Plant Breeding Research, Department of Plant Breeding and Genetics, Carl von Linné Weg 10, 50829 Cologne, Germany

**Keywords:** Cytoplasmic genome, Cytoplasmic male sterility, Molecular marker-assisted selection, Late blight resistance, Agronomic trait, Potato (*Solanum tuberosum* L.)

## Abstract

**Background:**

Various wild species germplasm has been used in European potato breeding since the first introduction of potato (*Solanum tuberosum* L.) to Europe. As the plant cytoplasmic genome including chloroplast and mitochondrial genomes is transmitted only through the maternal parent, cytoplasmic markers are useful tools in breeding programs to determine cytoplasmic genome types and to trace maternal ancestors. The potato cytoplasmic genome can be distinguished into six distinct types (M, P, A, W, T, and D). Male sterility was found in genotypes with *S. demissum*-derived D-type cytoplasm and *S. stoloniferum*-derived W/γ-type cytoplasm. These wild species were frequently used to incorporate superior pathogen resistance genes. As a result, the percentage of these two types is increasing unintentionally in the European germplasm pool. Other than cytoplasmic male sterility, little is known about effects of the cytoplasmic genome on complex agronomic traits in potato.

**Result:**

The cytoplasm types of 1,217 European potato cultivars and breeding clones were determined with type specific DNA markers. Most frequent were T- (59.4 %), D- (27.4 %), and W- (12.2 %) type cytoplasm, while A- (0.7 %) and M-type cytoplasm (0.3 %) was rare and P-type cytoplasm was absent. When comparing varieties with breeding clones, the former showed a relatively higher frequency of T-type and lower frequency of D- and W-type cytoplasm. Correlation analysis of cytoplasm types and agronomic data showed that W/γ-type cytoplasm was correlated with increased tuber starch content and later plant maturity. Correlation with quantitative resistance to late blight was observed for D-type and M-type cytoplasm. Both cytoplasm types had a positive effect on resistance.

**Conclusion:**

This study revealed and quantified the cytoplasmic diversity in the European potato germplasm pool. Knowledge of cytoplasm type is important for maintaining genetic diversity and managing the male sterility problem in breeding programs. This is the first comprehensive study to show correlations of distinct cytoplasmic genomes with complex agronomic traits in potato. Correlations particularly with tuber starch content and resistance to late blight provided new knowledge on cytoplasmic effects on these important traits, which can be exploited for genetic improvement of potato.

**Electronic supplementary material:**

The online version of this article (doi:10.1186/s12870-015-0545-y) contains supplementary material, which is available to authorized users.

## Background

In a plant cell, multiple copies of chloroplast and mitochondrial DNA co-exist with one copy of nuclear DNA. Thus, the expression and function of nuclear genes should be affected in various ways by chloroplast and mitochondrial genes. A typical phenomenon caused by an interaction between nuclear and mitochondrial genes is cytoplasmic male sterility frequently found in crop species [1]. Chloroplast and mitochondrial DNA compose the cytoplasmic genome and are maternally inherited in most angiosperms [2, 3]. Various effects of different cytoplasmic genomes on agronomic traits have been demonstrated in classical work in *Triticum* and *Aegilops* using cytoplasm substitution lines [4].

Potato (*Solanum tuberosum* L.) is a crop that possesses different types of cytoplasmic genomes within a cultivar group. Since its first introduction into Europe in the sixteenth century, many diseases have threatened potato cultivation. In order to overcome them, new genetic resources have been frequently utilized from cultivated Andean landraces and wild potato species [5, 6]. The first introduction into Europe is thought to have been *S. tuberosum* L. ssp. *andigena* (referred to as *S. tuberosum* L. Andigenum Group by Spooner *et al.* [7]. In this article we tentatively use the taxonomy of Hawkes [8]). Later Chilean forms (*S. tuberosum* L. ssp. *tuberosum*) were introduced which became the ancestors of modern cultivars improved for short stolons, early vine maturity and high tuber yield in Europe and North America [5, 6, 8, 9]. The first dramatic change in the cultivar spectrum happened with the late blight epidemics caused by *Phytophthora infestans*, resulting in the Irish Famine (1845–1847). Soon after the Famine, modern potato breeding started using only a few Chilean ssp. *tuberosum* clones including the cultivar Rough Purple Chili and an ancestor of Alte Daber [5, 6, 10]. In 1906, Salaman [11] detected resistance to late blight in *S.* × *edinense* Berth. and crossed it with ssp*. tuberosum*. In 1908, the Mexican hexaploid species *S. demissum* Lindl. was introduced, which then started the breeding for late blight resistance with *S. demissum* germplasm in Germany [5]. The resultant pentaploid hybrid could be backcrossed easily with *S. tuberosum*. More beneficial tetraploid hybrids were obtained by crossing the diploid cultivated species *S. phureja* Juz. et Buk. with *S. demissum* [5, 12]. These efforts resulted in more than 80 % of modern cultivars of Germany carrying genes from *S. demissum* [5]. Many aphid transmitted viruses also damage severely potato cultivation. Plants infected by *Potato virus Y* (PVY) and *Potato virus X* (PVX) are badly harmed. In Europe, extreme resistance to PVY was first detected by Stelzer in *S. stoloniferum* Schltdl. [13]. A dominant major gene *Ry*_*sto*_ was found by Ross [14] and Cockerham [15]. A major gene *Rx*_*acl*_ for resistance to PVX was found in *S. acaule* Bitt. [15, 16]. These resistance genes, sometimes found in complex hybrids such as those from a cross (*S. acaule* × *S. stoloniferum*) × *S. tuberosum*, were introduced into many parental lines in Germany. Potato cyst nematodes have raged throughout Europe probably since the mid-nineteenth century. The gene *H1* for extreme resistance to the potato cyst nematode *Globodera rostochiensis* (Woll.) was detected in ssp. *andigena* accession CPC 1673 in the Commonwealth Potato Collection [17–19]. Resistance to *G. pallida* pathotype Pa2/3 was introgressed into breeding lines from the wild potato species *S. vernei* Bitt. et Wittm. and *S. spegazzinii* Bitt.. Nowadays, most modern German cultivars have one or more nematode resistance genes from these species. After a seriously raging wart epidemic in 1910, caused by the fungus *Synchytrium endobioticum*, genes for resistance to *S. endobioticum* were found in some wild species [20, 21] and have been used for a long time for successful prevention [22, 23]. As briefly summarized above, resistance breeding in Europe has an intricate history and is based on the frequent use of wild species germplasm. Consequently, various cytoplasmic genomes are expected to be present in European potatoes. However, it is difficult to identify cytoplasmic genomes by tracing back the maternal lineage, often because no information is available about which parent was used as the female parent in a cross. Sometimes also no pedigree record is available for breeding clones.

Comparing reciprocal hybrid populations, it has long been known that the cytoplasm of *S. tuberosum* ssp. *tuberosum* is different from that of *S. tuberosum* ssp. *andigena*, the former inducing higher percentage of tuberization, higher tuber yield, higher tuber numbers, and earlier vine maturity [24–28]. Several cytoplasmic genomes were distinguished among cultivated potatoes and its related wild species [29]. Polymerase chain reaction (PCR)-based markers were developed that distinguish *S. tuberosum* ssp*. tuberosum*-type chloroplast DNA from the other chloroplast DNA types [30, 31]. Lössel *et al.* [30] also developed PCR markers that distinguish three mitochondrial DNA types (α-, β-, and γ-types). Recently, Hosaka and Sanetomo [32] developed a simpler and more informative technique using a set of five cytoplasmic markers (four chloroplast and one mitochondrial DNA markers), which differentiate six potato cytoplasm types: M, P, A, W, T, and D. The P- and A-type cytoplasm and the T- and D-type cytoplasm are relatively distinct types within the M- and W-type cytoplasm, respectively, each of which has diverse cytoplasmic variations [33, 34]. Andean cultivated potatoes evolved from ancestral wild species with M or M-derived type cytoplasm, while all other wild species not involved in the origin of cultivated potatoes have W or W-derived type cytoplasm [32]. The A-type cytoplasm is most prevalent in *S. tuberosum* ssp. *andigena*, while the T-type cytoplasm is most prevalent in *S. tuberosum* ssp. *tuberosum*. The P-type cytoplasm was introduced from *S. phureja* [35], while the D-type cytoplasm was introduced from *S. demissum* into the common potato gene pool [36]. This cytoplasm type definition system is validated only among cultivated potatoes and their close wild relatives [37]. Many wild species have specific cytoplasmic genomes [34], all of which are assigned as W-type cytoplasm. In order to distinguish the *S. stoloniferum-* derived cytoplasm carried by many modern varieties, an additional mitochondrial marker ALM_4/ALM_5, developed by Lössel *et al.* [30] is needed, by which three mitochondrial types, α-, β-, and γ-types can be distinguished. The *S. stoloniferum*-derived cytoplasm is characterized as W/γ subtype [30].

Cytoplasmic male sterility was found in interspecific crosses in potato, realizing the existence of different cytoplasm, as has been known in other crop species [1, 38, 39]. The common potato cytoplasm induces various types of intrinsic sterility [40]. Cultivars carrying the PVY resistance gene *Ry*_*sto*_ exhibit complete male sterility caused by interaction with mitochondrial DNA of *S. stoloniferum* [41, 42], which is characterized as W/γ subtype cytoplasm [30, 43]. Sterile pollen grains clumped together in tetrads, so it was called “tetrad sterility” [44] or “lobed sterility” [45]. The same type of sterility was also observed with *S. verrucosum*-derived cytoplasm [44]. Pentaploid F_1_ hybrids can be easily obtained when *S. demissum* is crossed as a female parent with *S. tuberosum*. The hybrids produce normal-looking pollen, however, they are non-functional as male parents. The progeny produced by continued backcrossing with the pollen of *S. tuberosum* can be used only as female parents, although these plants produce abundant stainable pollen [46]. Thus, the *S. demissum*-derived cytoplasm is also associated with functionally male sterile pollen. Once *S. stoloniferum* or *S. demissum* cytoplasm are incorporated into parental lines, they can be used only as female parents. Continued infiltration of the potato gene pool by these cytoplasm would worsen male sterility problems as warned by Provan *et al.* [47] and Hosaka and Sanetomo [32].

Marker-assisted selection is an efficient breeding tool that connects genotypes with agronomic traits and pathogen resistances. Various diagnostic DNA markers are available now for potato breeding [35, 48–50]. Recently, association genetics has been applied to identify diagnostic markers for quantitative traits that are controlled by multiple genes and environmental factors. For example, associations were discovered between DNA polymorphisms at individual candidate loci and complex traits such as tuber yield, starch and sugar content [51–54]. Gebhardt *et al.* [55] genotyped a gene bank collection of 600 potato cultivars with five DNA markers linked to a previously mapped quantitative trait locus (QTL) for resistance to late blight and plant maturity. Significant association with quantitative resistance to late blight and plant maturity was detected with PCR markers derived from *R1*, a major gene for race specific resistance to late blight, or tightly linked to *R1*. The marker alleles associated with increased resistance and later maturity were traced to an introgression from *S. demissum* [55]. Pajerowska-Mukhtar *et al.* [56] tested 24 candidate loci for association with field resistance to late blight and plant maturity in a population of 184 breeding clones and found single nucleotide polymorphisms (SNPs) in the *Allene Oxide Synthase 2* (*StAOS2*) gene associated with field resistance to late blight.

Using cytoplasmic markers, Lössel *et al.* [30] indicated that W/α and W/γ-type cytoplasm showed a higher tuber starch content than T/β-type cytoplasm. Apart from that, little is known about effects of the cytoplasmic genome on agronomic performance, mainly because an accurate method to distinguish cytoplasmic genomes was not available until recently.

In this study, we analyzed 1,383 tetraploid genotypes of six different populations to disclose the cytoplasmic diversity in European potato gene pool. These populations have been previously evaluated for agronomic traits such as late blight resistance, chip quality, tuber yield and starch content, plant maturity and susceptibility to tuber bruising in the context of searching for associations with nuclear markers. Correlations were investigated between different cytoplasm types and agronomic traits. The importance of cytoplasmic diversity and the correlation with some agronomic traits, especially with tuber starch content and resistance to late blight are discussed.

## Results

### Cytoplasm types of European potato collections

A total of 1,383 tetraploid cultivars and breeding clones of six populations (Table 1) were genotyped using multiplex PCR with the cytoplasmic markers T, S, SAC, D, and A. One genotype of population PIN184 showed a mixed pattern of T- and M-type cytoplasm, while another genotype of population CHIPS-ALL showed a mixed pattern of T- and D-type cytoplasm, probably due to DNA contaminations. These two genotypes were discarded for the further analysis. Genotypes with W-type cytoplasm were further examined using the mtDNA (mitochondrial DNA) marker ALM_4/ALM_5 which distinguished four different subtype cytoplasm: W/α, W/β, W/γ, and the fourth type. The fourth type detected in one cultivar had both 2.4 kb and 1.6 kb bands (= Type 3 banding pattern reported by Hosaka and Sanetomo [32]), which is designated as W/αβ-type cytoplasm in this article.

**Table 1:** Populations used and quantitative agronomic trait data evaluated previously

Population	Varieties	Breeding clones	Total	Traits evaluated^a^	Reference
BRUISE	85	120	205	BI, SCB, PM, TS, TSC, TY	[52]
CHIPS-ALL	34	194	228	CQA, CQS, TSC, TY, TSY	[50, 51, 54]
EURO-CUL	187	3	190	-	[83]
GBC	511	25	536	PM, RLBF, RLBT	[55]
PIN184	-	184	184	PM, rAUDPC, MCR, TSC	[56]
SUGAR40	39	1	40	RSC	[53]

^a^BI, bruising index; SCB, starch corrected bruising; PM, plant maturity; TS, tuber shape; TSC tuber starch content; TY, tuber yield; TSY, starch yield (=TSC × TY); CQA, chip quality after harvest; CQS, chip quality after 3 months storage at 4 °C; RLBF, foliage resistance to late blight; RLBT, tuber resistance to late blight; rAUDPC, the relative area under disease progress curve for the field infestation of *Phytophthora infestan*s; MCR, maturity corrected resistance to late blight; RSC, tuber reducing sugar content

The T-type cytoplasm was the most prevalent in all six populations (Table 2, Additional file 1: Table S1). The GBC population consisting of 536 genotypes included many old varieties. 369 varieties and 6 breeding clones had T-type cytoplasm, being the highest frequency of T-type cytoplasm (70.0 %) among all populations. On the other hand, T-type cytoplasm was found in less than half of the genotypes (45.9 %) in the PIN184 population, which represented modern breeding materials. In contrast, D-type cytoplasm was found with the highest frequency in the PIN184 population (35.5 %) and the lowest frequency in the GBC population (20.3 %). Within W-type cytoplasm, the subtype W/γ was the most frequent. The frequency of W/γ-type cytoplasm in the EURO-CUL and GBC populations was 11.6 % and 6.0 %, respectively, which was lower than those of the other four populations (13.7 % − 25.0 %). The variety “Raisa” and two breeding clones “CIP 38 31 17 06” and “MPI79.452/14D” had the W/α-type cytoplasm. Eighteen genotypes had the W/β-type. Only the variety “Rita”, which was included in both EURO-CUL and GBC populations, had W/αβ-type cytoplasm. A-type cytoplasm was found in four genotypes in EURO-CUL, five genotypes in GBC and one genotype in PIN184. All four genotypes with M-type cytoplasm were from the PIN184 population.

**Table 2:** The number of genotypes and percentages with different cytoplasm types in each population

Population	T	D	A	M	W/α	W/β	W/γ	W/αβ	Total
BRUISE	105 (54)^a^	69 (21)	0	0	0	1 (1)	30 (9)	0	205
	51.2 %	33.7 %	0.0 %	0.0 %	0.0 %	0.5 %	14.6 %	0.0 %	
CHIPS-ALL	114 (13)	78 (17)	0	0	0	3	32 (4)	0	227
	50.2 %	34.4 %	0.0 %	0.0 %	0.0 %	1.3 %	14.1 %	0.0 %	
EURO-CUL	118 (118)	42 (40)	4 (4)	0	1 (1)	2 (1)	22 (22)	1 (1)	190
	62.1 %	22.1 %	2.1 %	0.0 %	0.5 %	1.1 %	11.6 %	0.5 %	
GBC	375 (369)	109 (99)	5 (5)	0	2	12 (12)	32 (25)	1 (1)	536
	70.0 %	20.3 %	0.9 %	0.0 %	0.4 %	2.2 %	6.0 %	0.2 %	
PIN184	84	65	1	4	0	4	25	0	183
	45.9 %	35.5 %	0.5 %	2.2 %	0.0 %	2.2 %	13.7 %	0.0 %	
SUGAR40	22 (21)	8 (8)	0	0	0	0	10 (10)	0	40
	55.0 %	20.0 %	0.0 %	0.0 %	0.0 %	0.0 %	25.0 %	0.0 %	
Total without duplicates	723	333	9	4	3	18	126	1	1217
	59.4 %	27.4 %	0.7 %	0.3 %	0.2 %	1.5 %	10.4 %	0.1 %	

^a^The number of cultivars is shown in parentheses

One hundred and nineteen varieties and one breeding line were duplicated in at least two populations. Of these duplicated genotypes, 104 had the identical cytoplasm type in all duplicates, whereas 16 (13 %) had different cytoplasm types (Additional file 2: Table S2), probably due to sampling errors. Discarding these duplicates, a total of 1,217 varieties and breeding clones were actually determined for the cytoplasm types: 723 (59.4 %) had T-type, 333 (27.4 %) D-type, 9 (0.7 %) A-type, 4 (0.3 %) M-type and 148 (12.2 %) had W-type cytoplasm (Table 2). None of the genotypes had the P-type cytoplasm. Of 1,217 genotypes, 694 were named cultivars, of which 480 (69.2 %) had T-type, 148 (21.3 %) D-type, 8 (1.2 %) A-type and 58 (8.4 %) had W-type cytoplasm (W/γ = 6.5 %). The remaining 523 genotypes were breeding clones, of which 243 (46.5 %) had T-type, 185 (35.4 %) D-type, 1 (0.2 %) A-type, 4 (0.8 %) M-type and 90 (17.2 %) had W-type cytoplasm (W/γ = 15.5 %). The cultivars “Tannenzapfen” and “Pink Fir Apple” and the three cultivars “Asparges”, “Corne de Bique” and “La Ratte” were treated as different cultivars, but are suspected to be identical, according to the German potato gene bank at Groß-Lüsewitz and the Potato Pedigree Database at Wageningen [57]. All five cultivars had the A-type cytoplasm in common. The cytoplasm types of all 694 European varieties, 26 named breeding clones and 497 breeding clones with ID number of each breeding company are listed in Additional file 1: Table S1.

### Correlation of different cytoplasm types with quantitative agronomic traits

The different cytoplasm types were tested for correlation with 20 quantitative agronomic traits that have been evaluated in five of the six populations using one-way ANOVA (parametric) or Welch’s test (nonparametric) (Table 3). The cytoplasm type found in less than three genotypes in each population (Table 2) was omitted because it was a number too small to apply statistical analysis by ANOVA or Welch’s test.

**Table 3:** One-way ANOVA or Welch’s test of cytoplasm types with phenotypic scores for agronomic traits

Population	Trait^a^	Cytoplasm type	No. of genotypes	Mean (SD)	Statistics	
					F ratio^b^	Level^c^
BRUISE	BI	T	104	31.0 (2.05)	*F* = 6.13**	a
	Resistant (0) − highly susceptible (100)	D	69	36.5 (2.51)		ab
		W/γ	30	46.6 (3.81)		b
	SCB	T	104	−1.87 (1.469)	*F* = 1.57	
	Lower value indicates higher resistance	D	69	−0.95 (1.804)		
		W/γ	30	2.63 (2.736)		
	PM	T	104	3.49 (0.102)	*F* = 5.14**	a
	Very early (1) − very late (9)	D	68	3.47 (0.127)		a
		W/γ	30	4.38 (0.191)		b
	TS	T	104	3.79 (0.084)	*F* = 5.64**	a
	Completely circular (1) − longitudinal (9)	D	69	3.56 (0.103)		a
		W/γ	30	3.14 (0.156)		b
	TSC	T	104	15.0 (0.23)	*F* = 5.89**	a
	% fresh weight	D	69	15.7 (0.29)		ab
		W/γ	30	16.7 (0.43)		b
	TY	T	96	489.8 (7.36)	*F* = 3.63*	a
	dt/ha	D	59	512.1 (9.39)		ab
		W/γ	26	528.0 (14.14)		b
CHIPS-ALL	CQA	T	114	6.22 (0.187)	*F* = 0.24	
	Very dark (1) − very light (9)	D	78	6.03 (0.226)		
		W/β	3	6.27 (1.155)		
		W/γ	32	5.96 (0.354)		
	CQS	T	114	2.36 (0.206)	*F* = 0.77	
	Very dark (1) − very light (9)	D	78	2.54 (0.249)		
		W/β	3	2.55 (1.269)		
		W/γ	32	3.08 (0.389)		
	TSC	T	114	16.4 (0.29)	*F* = 3.77*	a
	% fresh weight	D	78	16.8 (0.35)		a
		W/β	3	16.6 (1.79)		ab
		W/γ	32	18.5 (0.55)		b
	TY	T	114	660.4 (9.06)	*F* = 1.71	
	dt/ha	D	78	637.0 (10.95)		
		W/β	3	645.2 (55.85)		
		W/γ	32	622.2 (17.10)		
	TSY	T	114	107.8 (2.21)	*F* = 0.94	
	=TSC × TY	D	78	106.2 (2.67)		
		W/β	3	106.4 (13.62)		
		W/γ	32	114.4 (4.17)		
GBC	PM	T	365	4.35 (0.096)	*F* = 2.70	
	Very late (1) − very early (9)	D	109	4.04 (0.175)		
		A	5	2.80 (0.817)		
		W/β	12	4.00 (0.527)		
		W/γ	32	4.06 (0.323)		
	RLBF	T	317	4.80 (0.093)	*F* = 8.71***	a
	Highly susceptible (0) − highly resistant (9)	D	95	5.76 (0.171)		b
		A	4	3.25 (0.831)		a
		W/β	12	5.83 (0.480)		b
		W/γ	25	5.84 (0.332)		b
	RLBT	T	287	5.49 (0.100)	*F* = 1.00	
	Highly susceptible (0) − highly resistant (9)	D	84	5.67 (0.185)		
		A	4	3.25 (0.847)		
		W/β	11	5.18 (0.511)		
		W/γ	23	5.70 (0.353)		
PIN184	MCR	T	84	0.03 (0.007)	*F* = 11.94***	a
	Lower value indicates higher resistance	D	65	−0.02 (0.008)		b
		M	4	−0.13 (0.033)		c
		W/β	4	0.01 (0.033)		ab
		W/γ	25	0.03 (0.013)		a
	rAUDPC	T	84	0.42 (0.009)	*F* = 11.53***	a
	Lower value indicates higher resistance	D	65	0.34 (0.010)		bc
		M	4	0.25 (0.039)		c
		W/β	4	0.32 (0.039)		abc
		W/γ	25	0.38 (0.016)		ab
	PM	T	84	5.82 (0.139)	*F* = 8.44***	a
	Very late (1) − very early (9)	D	65	5.39 (0.158)		bc
		M	4	6.82 (0.637)		ab
		W/β	4	3.73 (0.637)		d
		W/γ	25	5.00 (0.255)		b
	TSC	T	84	16.0 (0.29)	*F* = 7.25***	a
	% fresh weight	D	65	17.0 (0.33)		ab
		M	4	15.2 (1.32)		ab
		W/β	4	21.8 (1.32)		c
		W/γ	25	17.9 (0.53)		bc
SUGAR40	RSC	T	8	1.36 (0.355)	*F* = 1.39	
	% dry weight	D	22	0.60 (0.214)		
		W/γ	10	0.96 (0.318)		

^a^See Table 1 for abbreviations of traits

^b^ *, **, and ***: Significance levels at 5 %, 1 %, and 0.1 %, respectively. ANOVA test was performed for TY, TSC, TSY, SCB, rAUDPC and MCR and Welch’s test for BI, PM, TS, CQA, CQS, PM, RLBE, RLBT, and RSC

^c^Means of each pair were compared using Tukey’s test or Kruskal-Wallis test. Means that are not sharing the same alphabets are significantly different at the 5 % level

For the significant traits, means of each pair were compared using Tukey’s test performed after ANOVA or Kruskal-Wallis test performed after Welch’s test.

In the BRUISE population (*n* = 203), T-, D- and W/γ-type cytoplasm were tested for correlation with the phenotypic traits BI (bruising index), SCB (starch corrected bruising), PM (plant maturity), TS (tuber shape), TSC (tuber starch content), and TY (tuber yield). For five of the six traits significant differences among cytoplasm types were found (Table 3). Starch corrected bruising was not significantly different among cytoplasm types. Genotypes with W/γ-type cytoplasm matured significantly later, and had more round tuber shape compared to those with T- or D-type cytoplasm. Compared to genotypes with T-type cytoplasm, genotypes with W/γ-type cytoplasm were more susceptible to black spot bruising, had higher tuber starch content and yield.

Chip quality was analyzed as CQA (chip quality after harvest) and CQS (chip quality after 3 months storage at 4 °C) in the CHIPS-ALL population (*n* = 227) and as RSC (tuber reducing sugar content) in the SUGAR40 population (*n* = 40). No significant difference was found among cytoplasm types for the three chip quality traits. Of the traits TSC, TY and TSY (tuber starch yield) analyzed in the CHIPS-ALL population, only TSC showed significant differences between cytoplasm types. Consistent with the observations in the BRUISE population, TSC was significantly higher in genotypes with W/γ-type cytoplasm compared to those with T- or D-type cytoplasm.

In the GBC population (*n* = 536), passport data for RLBF (foliage resistance to late blight) and RLBT (tuber resistance to late blight) were analyzed for correlation with cytoplasm types. Welch’s test revealed a significant difference for RLBF. Genotypes with T- and A-type cytoplasm showed lower resistance levels compared to those with D-, W/γ and W/β-type cytoplasm. PM did not show significant differences among cytoplasm types.

In the PIN184 population (*n* = 182), T, D, M, W/β and W/γ cytoplasm types were analyzed for correlation with resistance to late blight measured as MCR (maturity corrected resistance) and rAUDPC (relative area under disease progress curve). The results showed that cytoplasm type had a clearly significant effect on resistance to late blight (Table 3). Genotypes with D-type cytoplasm showed a significantly lower mean MCR value compared to those with T or W/γ-type cytoplasm (Table 3). The four genotypes with M-type cytoplasm had the lowest mean MCR value (−0.13). Thus, both M- and D-type cytoplasm were correlated with increased resistance to late blight. The same was true for rAUDPC, which showed higher resistance levels to late blight with M- and D-type cytoplasm compared to those with T-type cytoplasm. Mean PM and TSC also differed significantly among genotypes with different cytoplasm types. Genotypes with W/β-type cytoplasm had the latest maturity but the highest tuber starch content (21.8 %) compared to those with the other cytoplasm type. Genotypes with W/γ-type cytoplasm matured later and also had higher starch content (17.9 %) compared to those with T-type cytoplasm (16.0 %).

Thus, compared with the T-type cytoplasm, the D-type cytoplasm was correlated with increased foliage resistance to late blight in two independent populations (GBC and PIN184). The W/γ-type cytoplasm was correlated with higher tuber starch content in three populations (BRUISE, CHIPS-ALL and PIN184) and with later maturity in two populations (BRUISE and PIN184).

### Correlation of D-type cytoplasm with nuclear gene markers for late blight resistance

The fact that genotypes with D-type cytoplasm showed a higher average level of late blight resistance compared to those with T-type cytoplasm in both the GBC and PIN184 populations (Table 3), could result from the joint introgression of D-type cytoplasm with *R* genes from *S. demissum.* We tested therefore whether the presence of the marker diagnostic for D-type cytoplasm was correlated with the presence of nuclear markers closely linked or identical with the late blight resistance genes *R1*, *R3a* and *R3b*.

Correlation coefficients were obtained for the D-type cytoplasmic marker with four markers either located in the *R1* resistance gene (R1_1400_, R1_1800_) or tightly linked to *R1* (CosA_210_ and GP179_570_) that have been scored in the GBC population [55], and with one *R1* diagnostic marker (CosA_210_), and *R3a* and *R3b* gene specific markers scored in the PIN184 population [56]. None of nuclear markers for *R* genes showed significant correlation with the D cytoplasmic marker neither in the GBC nor the PIN184 population (Table 4).

**Table 4:** *Pearson*’s correlation coefficients between D-type cytoplasm and the nuclear markers linked with resistance genes *R1*, *R3a* and *R3b*

Population	Nuclear marker	Correlation coefficient (*r*)^a^
GBC	R1_1400_	0.23
	R1_1800_	−0.08
	CosA_210_	0.22
	GP179_570_	−0.11
PIN184	CosA_210_	0.06
	R3a	0.16
	R3b	0.27

^a^Correlation coefficient (r) was analyzed for the D-type cytoplasm with four markers in the GBC population, and with three markers in the PIN184 population.

Furthermore, the frequencies of the *StAOS2_A*_*691*_*C*_*692*_ haplotype were analyzed with different cytoplasm types by a Welch’s test in the PIN184 population (Table 5). The *StAOS2_A*_*691*_*C*_*692*_ haplotype was associated with higher late blight resistance [56]. Significant difference was found at a 5 % level. Kruskal-Wallis test indicated that the genotypes with D- and M-type cytoplasm had higher haplotype frequencies compared to those with T-type cytoplasm.

**Table 5:** Welch’s test for cytoplasmic differences on allele frequencies of SNP haplotype *StAOS2_A*_*691*_ *C*_*692*_ in the PIN184 population

Cytoplasm type	No. of genotypes	Allele frequency (SD)	F ratio^a^	Level^b^
T	82	0.40 (0.235)	*F* = 3.43*	a
D	63	0.55 (0.285)		b
M	4	0.69 (0.239)		b
W/β	4	0.56 (0.375)		ab
W/γ	25	0.49 (0.310)		ab

^a^ *: Significance level at 5 %

^b^Means of each pair were compared using Kruskal-Wallis test. Means that are not sharing the same alphabets are significantly different at the 5 % level

### Combined effects of nuclear markers and D-type cytoplasm on late blight resistance and plant maturity

Since no correlation was found between *R* gene nuclear markers and D-type cytoplasm, their combined effects on late blight resistance and plant maturity were evaluated. Four marker classes +/+, +/−, −/+, and −/− were tested by Welch’s test for significant differences, where ‘plus’ indicates the presence and ‘minus’ the absence of the marker, irrespective of allele dosage.

In the GBC population, the four marker classes of all combinations of nuclear markers with D-type cytoplasm differed highly significantly for RLBF (*P* < 0.001) (Table 6). Two-way ANOVA was performed using the nuclear markers and D-type cytoplasm as two contributing factors. Only D-type cytoplasm was significant (*P* < 0.001), indicating that genotypes with the D-type cytoplasm were more resistant to foliage late blight. Interaction of the D-type cytoplasm with GP179_570_ was found at the 5 % significance level. For RLBT, the marker classes combining nuclear markers R1_1400_, R1_1800_ and CosA_210_ with the D-type cytoplasm were significantly different. However, only the nuclear markers were significant factors. The presence of R1_1400_ and CosA_210_ indicated higher resistance to tuber late blight, while the presence of R1_1800_ indicated lower resistance to tuber late blight. For PM, significant difference was not found in any nuclear marker and D-type cytoplasm combination.

**Table 6:** Interaction for effects of combinations of presence (+) and absence (−) of nuclear markers with presence (+) or absence (−) of D-type cytoplasm on resistance to late blight and plant maturity in the GBC population. In case differences were found among marker classes, two-way ANOVA was performed to explore the factors contributing the differences

Marker combination	Marker class	Foliage resistance to late blight (RLBF)				Tuber resistance to late blight (RLBT)				Plant maturity (PM)			
		n	Mean (SD)	Welch’s test	Two-way ANOVA	n	Mean (SD)	Welch’s test	Two-way ANOVA	n	Mean (SD)	Welch’s test	Two-way ANOVA
R1_1400_/D	+/+	48	5.9 (1.52)	***	D***	44	5.8 (1.42)	**	R1_1400_*	53	3.7 (1.84)	ns	
	−/+	41	5.7 (1.52)			35	5.6 (1.87)			46	4.3 (2.09)		
	+/−	94	5.3 (1.78)			81	6.1 (1.55)			104	4.0 (1.75)		
	−/−	233	4.8 (1.72)			218	5.3 (1.77)			268	4.3 (1.82)		
R1_1800_/D	+/+	41	5.6 (1.63)	***	D***	38	5.6 (1.72)	**	R1_1800_*	46	3.8 (1.99)	ns	
	−/+	43	6.1 (1.37)			37	5.9 (1.41)			48	4.1 (2.02)		
	+/−	180	4.8 (1.77)			164	5.3 (1.91)			207	4.1 (1.73)		
	−/−	129	5.3 (1.71)			114	6.0 (1.34)			145	4.5 (1.91)		
CosA_210_/D	+/+	48	5.9 (1.57)	***	D***	43	5.8 (1.40)	**	CosA_210_*	56	3.8 (1.78)	ns	
	−/+	44	5.7 (1.53)			39	5.5 (1.79)			50	4.2 (2.21)		
	+/−	94	5.4 (1.69)			82	6.0 (1.61)			104	4.0 (1.72)		
	−/−	257	4.8 (1.71)			236	5.3 (1.75)			302	4.4 (1.81)		
GP179_570_/D	+/+	40	6.2 (1.34)	***	D***	35	6.0 (1.29)	ns		43	4.1 (1.91)	ns	
	−/+	52	5.5 (1.63)		GP179_570_ × D*	47	5.5 (1.77)			62	3.9 (2.07)		
	+/−	188	4.8 (1.72)			171	5.4 (1.78)			219	4.2 (1.63)		
	−/−	162	5.0 (1.74)			146	5.6 (1.69)			185	4.5 (1.95)		

*, **, and ***indicate significance levels at 5 %, 1 %, and 0.1 %, respectively. ns, not significant

In the PIN184 population, mean MCR and rAUDPC were both significantly different among marker classes combining D-type cytoplasm with either R3a, R3b, or CosA_210_ markers (Table 7). By two-way ANOVA, D-type cytoplasm was the significantly contributing factor in these combinations and indicated a higher level of resistance. Some additional effects of interactions of D-type cytoplasm with CosA_210_ for MCR were detected. Combination of D-type cytoplasm with CosA_210_ affected PM. D-type cytoplasm and its interaction with CosA_210_ were contributing factors; the presence of D-type cytoplasm resulted in later maturity, and in combination with CosA_210_ resulted in the latest maturity, whereas the presence of CosA_210_ without D-type cytoplasm resulted in the earliest maturity. The dosage classes of the SNP haplotype *StAOS2_A*_*691*_*C*_*692*_ were grouped in two genotype classes, one lacking the haplotype *StAOS2_A*_*691*_*C*_*692*_ and the other with the *StAOS2 A*_*691*_*C*_*692*_ haplotype. The two *StAOS2* marker classes were combined with presence or absence of the D-type cytoplasm and analyzed for effects on MCR, rAUDPC, and PM (Table 7). Significant differences among marker classes were found for all traits, although the number of genotypes lacking haplotype *StAOS2_A*_*691*_*C*_*692*_ was small. By two-way ANOVA, StAOS2_SNP691/692 was found to be a significantly contributing factor. Haplotype *StAOS2 A*_*691*_*C*_*692*_ was more resistant to late blight and later maturing. The contribution of D-type cytoplasm was not detected. However, the combination of D-type cytoplasm with haplotype *StAOS2_A*_*691*_*C*_*692*_ showed significantly higher late blight resistance (MCR and rAUDPC) than *StAOS2_A*_*691*_*C*_*692*_ alone (*P* < 0.001), whereas no difference was observed for plant maturity (*P* = 0.42).

**Table 7:** Interaction for effects of combinations of presence (+) and absence (−) of nuclear markers with presence (+) or absence (−) of D-type cytoplasm on resistance to late blight and plant maturity in the PIN184 population. In case significant differences were found among marker classes, two-way ANOVA was performed to explore the factors contributing the differences

Marker combination	Marker class	Maturity corrected resistance (MCR)				Relative area under disease progress curve (rAUDPC)				Plant maturity (PM)			
		n	Mean (SD)	Welch’s test	Two-way ANOVA	n	Mean (SD)	Welch’s test	Two-way ANOVA	n	Mean (SD)	Welch’s test	Two-way ANOVA
R3a/D	+/+	32	−0.01 (0.077)	***	D***	32	0.37 (0.096)	***	D***	32	5.6 (1.26)	ns	
	−/+	33	−0.04 (0.071)			33	0.32 (0.076)		R3a*	33	5.2 (1.28)		
	+/−	39	0.02 (0.056)			39	0.40 (0.078)			39	5.8 (1.41)		
	−/−	79	0.03 (0.071)			79	0.40 (0.081)			79	5.5 (1.33)		
R3b/D	+/+	53	−0.02 (0.076)	***	D***	53	0.34 (0.092)	***	D***	53	5.5 (1.23)	ns	
	−/+	12	−0.02 (0.074)			12	0.33 (0.078)			12	4.9 (1.44)		
	+/−	64	0.01 (0.059)			64	0.39 (0.081)			64	5.8 (1.45)		
	−/−	54	0.04 (0.071)			54	0.41 (0.078)			54	5.4 (1.23)		
CosA_210_/D	+/+	16	−0.01 (0.062)	***	D*	16	0.33 (0.060)	***	D**	16	4.7 (0.98)	*	D**
	−/+	49	−0.03 (0.079)		CosA_210_ × D*	49	0.35 (0.097)			49	5.6 (1.29)		CosA_210_ × D**
	+/−	23	0.00 (0.077)			23	0.38 (0.092)			23	6.1 (1.30)		
	−/−	95	0.04 (0.062)			95	0.40 (0.076)			95	5.5 (1.35)		
StAOS2_SNP691/692 (AC haplotype)^a^/D	+/+	60	−0.03 (0.075)	***	StAOS2_SNP691/692**	60	0.33 (0.086)	***	StAOS2_SNP691/692***	60	5.3 (1.26)	**	StAOS2_SNP691/692**
	−/+	3	0.06 (0.017)			3	0.48 (0.026)			3	6.9 (1.09)		
	+/−	108	0.02 (0.066)			108	0.39 (0.077)			108	5.5 (1.31)		
	−/−	8	0.07 (0.076)			8	0.48 (0.059)			8	6.6 (1.16)		

^a^A genotype with adenine at position 691 and cytosine at position 692, irrespective of their dosages, was regarded as an AC haplotype

*, **, and ***indicate significance levels at 5 %, 1 %, and 0.1 %, respectively. ns, not significant

## Discussion

### Cytoplasmic diversity in European potatoes

We found that T (59.4 %), D (27.4 %) and W/γ (10.4 %) were the major cytoplasm types in 694 varieties and 523 breeding clones of European potatoes. Lössl *et al.* [30] analyzed 144 German varieties and 140 di-haploid breeding clones and found plastid-mitochondrial types- T/β (corresponding to T-type cytoplasm) in 47 %, W/α (corresponding to D-type cytoplasm) in 40 %, and W/γ-type cytoplasm in 10 % of the analyzed genotypes. Thus, our result when using a much larger number of genotypes supports the finding of Lössl *et al.* [30] that T-, D-, and W/γ-type cytoplasm in this order, were the most prevalent cytoplasm types in European potatoes. However the frequencies of the respective cytoplasm types differed considerably between the two studies, as well as between varieties and breeding clones and between populations analyzed in our study (Table 2).

Previously, a collection of 488 Japanese potato germplasm including 84 varieties, 378 breeding clones and 26 landraces was investigated for the cytoplasm types. T, D, P, A, M and W types were found with frequencies of 72.1 %, 17.8 %, 6.4 %, 1.2 %, 0.2 % and 2.3 %, respectively [32]. The Japanese collection seems essentially similar to European potatoes in the sense that T-type cytoplasm was the most prevalent. However, the frequencies of D- and W-type cytoplasm were much lower in the Japanese collection compared to European potatoes.

The T-type cytoplasm is understandably predominant in European and Japanese potatoes because most varieties maternally descended from ‘Rough Purple Chili’ and a few other clones from ssp*. tuberosum* [6, 47]. The differences in the frequencies of D- and W-type cytoplasm likely result from a more extensive use of *S. demissum*-derived late blight resistance and *S. stoloniferum*-derived PVY resistance in German breeding programs [5, 30, 32]. As chemical control has become a standard practice over the last few decades, late blight resistance breeding had lower priority in Japanese potato breeding [58]. In addition, the *S. chacoense*-derived PVY resistance gene *Ry*_*chc*_ has been used in Japanese breeding programs instead of *S. stoloniferum*-derived PVY resistance genes [35, 59]. For these reasons, the frequencies of D- and W/γ-type cytoplasm in Japanese potato germplasm are still lower compared to European potatoes. In contrast, the frequencies of D- and W-type cytoplasm were also much higher (38 % and 11 %, respectively, [60]) in the breeding program of the International Potato Center (CIP = Centro International de la Papa), because CIP aims to deliver pest- and disease-resistant varieties for developing countries where chemical control is not practical.

It is known that clones with the D- or W/γ-type cytoplasm are functionally male sterile [30, 41, 42, 44–46], so that these clones were used only as female parents, resulting in the infiltration of the common potato gene pool by these cytoplasm types [32]. The comparison of European, Japanese and Latin American gene pools demonstrates that our gene pools are being infiltrated by male sterility accompanying the D- or W/γ-type cytoplasm. The increasing frequencies of D- and W/γ- type cytoplasm enlarge the problem in designing successful mating combinations because the choice of male parents will be strictly limited, as warned previously by Provan *et al.* [47] and Hosaka and Sanetomo [32]. Another *S. stoloniferum*-derived PVY resistance gene *Ry-f*_*sto*_ could be used instead of *Ry*_*sto*_ because it is delivered through male fertile clones [61, 62]. Some genotypes with D-type cytoplasm also have been empirically known to be male fertile. Once the target gene(s) is transferred to genotypes with a cytoplasmic genome other than D- or W/γ-type cytoplasm, breeders could be liberated from tedious crossing activities associated with male sterility. Alternatively, a fertility-restoring gene, such as the *Rt* gene, which partially circumvents male sterility caused by the nuclear and T-type cytoplasm interactions [63], can be searched among genotypes with the D- or W/γ-type cytoplasm.

A prominent difference between European and Japanese potatoes was the presence of P-type cytoplasm (6.4 %) in the Japanese collection, while none was found in European potatoes. In Japan, the first diploid variety “Inca-no-mezame” was released in 2001, which has P-type cytoplasm derived from *S. phureja* [64]. It has an excellent taste, although it produces smaller tubers and lower yield than tetraploid cultivars. A chromosome-doubled, tetraploid clone with excellent taste was formed from “Inca-no-mezame” and combined with cyst nematode and PVY resistance genes, resulting in a breeding line “Saikai 35” [35]. Since good male fertility was recognized in genotypes with P-type cytoplasm, Saikai 35 has been extensively used to create new series of breeding clones for double cropping in Southern Japan [35]. This is why the P-type cytoplasm is increasingly present in Japanese breeding programs.

### Cytoplasmic origin of European potatoes

The T-type cytoplasm was most likely derived from Chilean *S. tubersoum* ssp. *tuberoum* via a series of selfed lines “Rough Purple Chili”, “Garnet Chili”, and “Early Rose”, and a few other clones [6, 10]. All D-type cytoplasm was derived from *S. demissum*, whilst W-type cytoplasm was designated to a diverse group of wild potato species [32]. Within W-type cytoplasm, α-, β-, and γ-type mitochondrial DNA can be distinguished using one PCR marker (ALM_4/ALM_5, [30]). Although the W/γ subtype was assigned to genotypes with *S. stoloniferum*-derived cytoplasm [30], the W/γ subtype was not only found in *S. stoloniferum* but also in *S. chacoense* Bitt., *S. pampasense* Hawkes, *S. pinnatisectum* Dun., and *S. vernei* Bitt. et Wittm. [32]. Fifty-two of 720 publicly available genotypes (Additional file 1: Table S1) had W/γ-type cytoplasm. According to the literature [5, 30, 43] at least 19 varieties and one MPI breeding clone had either the *S. stoloniferum*-derived *Ry*_*sto*_ gene or *S. stoloniferum* cytoplasm. Moreover, at least 21 of 25 breeding clones with W/γ-type cytoplasm in the PIN184 population were male sterile (H.-R. Hofferbert and E. Tacke, personal communication). Thus, most genotypes with W/γ-type cytoplasm were likely derived from *S. stoloniferum. S. stoloniferum* is a Mexican tetraploid species and is highly polymorphic [65], in which at least W/α-, W/γ-, and D-type cytoplasm were detected [30, 66, 67]. Nevertheless, varieties having the *Ry*_*sto*_ gene have exclusively W/γ-type cytoplasm and their maternal lineages could be traced back to three *S. stoloniferum* accessions from which parental lines MPI 13128, MPI 46.152/1 and 43/60/96/1 were derived [43]. It is unknown whether the W/γ-type cytoplasm interacts in some way with the nuclear gene *Ry*_*sto*_ for expression of resistance to PVY. Genotypes with another *S. stoloniferum*-derived PVY resistance gene *Ry-f*_*sto*_ are male fertile [61] and have D-type cytoplasm (unpublished data). Thus, it is a further question whether tetrad-sterility is caused by the W/γ-type cytoplasm of *S. stoloniferum* or by a few *S. stoloniferum* accessions introduced to the aforementioned parental lines of a source of *Ry*_*sto*_.

In the past, German potato breeding used *S. vernei* as a source for resistance to *Globodera rostochiensis* [5, 68, 69], implying that some genotypes may have W/γ-type cytoplasm derived from *S. vernei.* According to pedigree analysis, sixteen varieties might retain *S. vernei* germplasm. However, twelve of those varieties had T-type cytoplasm (cvs. Amethyst, Amigo, Aula, Benol, Compagnon, Culpa, Danva, Darwina, Hydra, Krostar, Puntila and Valetta), while the remaining four had D-type cytoplasm (cvs. Astarte, Mara, Nordlicht and Roxy) (Additional file 1: Table S1). This suggests that *S. vernei* was used as a pollen parent in the initial cross or its hybrid progeny of a later generation.

W/αβ-type cytoplasm was found in one variety “Rita”. This type has been found in *S. chacoense* Bitt. [32] and *S. spegazzinii* (Hosaka K, personal communication). *S. spegazzinii* was used in the past as a source for resistance to pathotypes Ro1 and Ro5 of *G. rostochiensis* [5]. Thus, “Rita” might have *S. spegazzinii*-derived cytoplasm.

Nine genotypes had the A-type cytoplasm, which was possibly derived from *S. tuberosum* ssp. *andigena*. Four genotypes had the M-type cytoplasm (Table 2). Considering German breeding history, *S. acaule* might be the most probable source for this cytoplasm, although pedigrees for these breeding clones are not available. As W/α- and W/β-type cytoplasm was found in various wild species [32], we could not clarify the cytoplasmic origin for the few genotypes with W/α- or W/β-type cytoplasm.

### Correlation of different cytoplasm types with agronomic traits

To the best of our knowledge, this study is the first that uses large populations to demonstrate a correlation of distinct cytoplasmic genomes with complex agronomic traits in potato. We found significant effects of cytoplasm type on various agronomic traits such as resistance to late blight and tuber bruising, plant maturity, tuber shape, starch content and yield (Table 3). No cytoplasmic difference was found for processing quality traits such as chip color and reducing sugar content (Table 3). In classical studies using reciprocal hybrids, it has been shown that the cytoplasmic genome of *S. tuberosum* ssp. *tuberosum* (mostly T-type cytoplasm) is characterized by higher percentage of tuberization, higher tuber yield, higher tuber numbers, and earlier vine maturity compared with that of *S. tuberosum* ssp. *andigena* (mostly A-type cytoplasm) [24–28]. Unfortunately, in the present study, genotypes with A-type cytoplasm were too rare for statistically significant comparisons between T- and A-type cytoplasm. Nevertheless, earlier plant maturity of genotypes with T-type cytoplasm compared to other cytoplasm types was observed in three independent populations (BRUISE, GBC, and PIN184), although statistical support was obtained only in the BRUISE and PIN184 populations (Table 3). A small effect on tuber yield was only observed in the BRUISE population.

### Correlation of W/γ-type cytoplasm with tuber starch content

W/γ-type cytoplasm was strongly correlated with increased tuber starch content in all three populations evaluated for TSC (Table 3). Lössl *et al.* [30] ranked tuber starch content according to W/γ (19 varieties) ≥ D (46 varieties) > T (79 varieties), which is in good agreement with our findings. Starch biosynthesis and degradation takes place in chloroplasts and amyloplasts and is controlled by a large number of nuclear genes. Eighteen quantitative traits loci (QTLs) for tuber starch-content were identified on all 12 potato linkage groups [70]. Efficiency of photosynthesis and carbon flux from source to sink tissues are important for the accumulation of starch in the tuber amyloplasts [71]. It is therefore conceivable that not only nuclear but also cytoplasmic factors play a functional role in starch accumulation and degradation and thereby cytoplasm type influences tuber starch content [30]. Higher tuber starch content is correlated with increased susceptibility to tuber bruising, later plant maturity and more circular tuber shape [52]. Thus, the effects of W/γ-type cytoplasm on the traits BI, PM and TS can be explained by correlation with TSC (Table 3).

### Correlation of cytoplasm type with late blight resistance

Data on late blight resistance were available for the GBC and PIN184 populations [55, 56]. The two populations were rather different. The GBC population consisted of historical varieties and breeding materials that were accompanied by resistance scores obtained in various environments. The PIN184 population represented contemporary germplasm of commercial breeding programs in Germany, and all genotypes were field evaluated under similar conditions. Nevertheless reproducible effects of cytoplasm type on foliage resistance to late blight were observed in both populations. Genotypes with D-type cytoplasm had on average higher levels of foliage resistance to late blight than genotypes with T-type cytoplasm. Genotypes with M-, W/β-, or W/γ-type cytoplasm also tended to have higher foliage resistance compared to those with T-type cytoplasm (Table 3). The four genotypes with M-type cytoplasm had the highest resistance level.

For the GBC population, Gebhardt *et al.* [55] reported that presence of the allele-specific markers CosA_210_, and R1_1400_ was associated with increased resistance of both foliage and tubers to late blight, whereas presence of R1_1800_ was associated with increased susceptibility. CosA_210_ and R1_1400_, but not R1_1800_, were also slightly associated with later maturity. The effect of D-type cytoplasm on late blight resistance was independent from the presence of the *R1* resistance gene on Chromosome V because none of the nuclear *R1* markers was correlated with D-type cytoplasm (Table 4). The combination of D-type cytoplasm with the nuclear *R1* markers showed that the effects of D-type cytoplasm was dominant (Table 6). This is probably because *S. demissum*-derived *R* genes other than *R1* were also incorporated together with D-type cytoplasm in the GBC population. D-type cytoplasm did not affect tuber resistance to late blight (Table 6). Consequently, D-type cytoplasm was not a contributing factor to tuber resistance in the marker combinations.

In the PIN184 population, neither *R1* nor *R3* markers showed association with late blight resistance [56]. Accordingly, in combinations of D-type cytoplasm with these markers only the D-type cytoplasm contributed to the observed significant effects on late blight resistance, confirming the independence of the effect of D-type cytoplasm from the *R* genes. The late blight resistance genes *R3a* and *R3b* are tightly linked with *StAOS2* (allene oxide synthase 2) on chromosome XI [56]. *StAOS2* was identified as a major locus for quantitative resistance to late blight in the PIN184 population and might also have a functional role in quantitative resistance to black leg/tuber soft rot caused by bacterium *Erwinia carotovora* ssp. *atroseptica* [56, 72, 73]. The frequencies of the SNP haplotype *StAOS2_A*_*691*_*C*_*692*_ which is associated with increased resistance were significantly higher in genotypes with M- and D-type cytoplasm compared to those with T-type cytoplasm (Table 5). In combination with D-type cytoplasm, the SNP haplotype *StAOS2_A*_*691*_*C*_*692*_ dominated the effect of the D-type cytoplasm. However, genotypes having both D-type cytoplasm and haplotype *StAOS2_A*_*691*_*C*_*692*_ had the highest level of resistance to late blight compared to the other three marker classes (Table 7), suggesting that the combination of both markers might improve field resistance to late blight more than each marker alone. The *StAOS2* gene encodes a key enzyme in the biosynthesis of the defense signaling molecule jasmonic acid [74]. Plant AOS enzymes are localized in chloroplasts [75–78]. Cloned *StAOS2* alleles included chloroplast targeting signal peptides and were localized in potato chloroplasts [73, 79]. In general, processes in the chloroplast seem to play a role in quantitative resistance to late blight, because many nuclear genes operating in the chloroplast show constitutive higher expression levels in genotypes with quantitative resistance when compared to more susceptible genotypes [80]. Cytoplasm type might interfere with these processes either positively or negatively, thus providing as explanation for the effects of cytoplasm type on resistance.

## Conclusions

Until recently, potato cytoplasmic genomes could be distinguished only by phenotypic comparisons between reciprocal cross hybrids. Molecular markers that could be used practically distinguished only between *S. tuberosum* ssp. *tuberosum*-type (T-type) and the other types [7, 9, 81, 82]. The new PCR-based technique distinguishes several potato cytoplasm types, and is simple, inexpensive and rapid, thereby facilitating large-scale cytoplasmic surveys in a short period of time [32]. Applying this technique to a large collection of European potatoes, we show that the use of M-, A- and P-type cytoplasm of Andean primitive cultivated potatoes was very limited in European potato breeding. Instead, the male-sterility-inducing D- and W/γ-type cytoplasm have been extensively used. The genetic basis of the cultivated potato could be broadened by introgression of cultivars with M- A- and P-type cytoplasm.

Thanks to the fact that phenotypic data for several important agronomic traits were available for the majority of the collection genotyped in this study for cytoplasm type, it was possible to assess for the first time whether cytoplasm type affects agronomic performance. D- and W/γ-type cytoplasm showed positive correlations with tuber starch content and foliage resistance to late blight. We identified four breeding clones with high resistance to late blight, which had the M-type cytoplasm. The M-type cytoplasm is a major cytoplasm among ancestral wild species of cultivated potatoes [34], but is rarely found in modern varieties [32]. The M-type cytoplasmic genome might have a direct function in resistance or is just co-inherited with other, unknown nuclear resistance factors. Identifying the cytoplasm types and verifying their relationships with phenotypic differences will contribute to the understanding of cytoplasmic genome diversity and function. Cytoplasm type is a novel DNA-based marker that can be used in combination with nuclear markers, for more efficient germplasm enhancement in potato.

## Methods

### Plant materials and phenotypic data

A total of 1,383 tetraploid genotypes consisting of 856 varieties and 527 breeding clones were determined for the cytoplasm type (Tables 1, 2 and Additional file 1: Table S1). These consisted of six populations, five of which have been used for association mapping: 205 genotypes selected for variation of susceptibility to tuber bruising [52] here referred to as the ‘BRUISE’ population, 228 genotypes selected for variation of chip quality [50, 51] referred to as the ‘CHIPS-ALL’ population, a gene bank collection of 536 genotypes with passport data for late blight resistance and plant maturity [55] referred to as the ‘GBC’ population, 40 genotypes selected for high and low processing quality [53] referred to as the ‘SUGAR40’ population, and 184 genotypes selected for variation of late blight resistance [56] referred to as the ‘PIN184’ population. The sixth population of 190 genotypes was named ‘EURO-CUL’ (European cultivars), included 140 varieties grown between 1987 and 1994 in the field at Scharnhorst (outstation of the MPI for Plant Breeding Research), 121 of which were used by Görg *et al.* [83] for variety identification, 10 varieties collected in the summer 2013 at the demonstration garden of the Max Planck Institute for Plant Breeding Research at Cologne, 29 varieties and three breeding clones received between 2001 and 2013 from the IPK Genebank, Germany, and the remaining 8 varieties obtained from various sources.

Evaluation data for several quantitative agronomic traits were available for these populations except EURO-CUL (Table 1). These phenotypic data were used in this study to evaluate effects of different cytoplasmic DNA types. The BRUISE population has been phenotyped for susceptibility to black spot bruising upon mechanical impact, measured as bruising index (BI) and starch corrected bruising (SCB), plant maturity (PM), tuber shape (TS), tuber starch content (TSC) and tuber yield (TY) [52]. The CHIPS-ALL population has been evaluated for chip quality after harvest (CQA) and after 3 months storage at 4 °C (CQS), TSC, tuber yield (TY) and starch yield (TSY = TSC × TY) [51]. Scores between 1 and 9 for plant maturity (PM), foliage resistance to late blight (RLBF) and tuber resistance to late blight (RLBT) were available for the GBC population [55]. This population has been genotyped with four nuclear DNA markers selected based on linkage to QTL for resistance to late blight and plant maturity for association analysis [55]. Markers CosA, and GP179, and the *R1* gene for resistance to late blight [84–86] are located within 1 cM on potato chromosome V in a “hot spot” for pathogen resistance [87]. Phenotypic traits available for the PIN184 population were the relative area under disease progress curve (rAUDPC) for field infestation of *Phytophthora infestan*s, plant maturity (PM) and maturity corrected resistance (MCR) [56]. In addition, the PIN184 population has been evaluated for tuber starch content (TSC) by measuring the specific gravity. Adjusted entry means for TSC across environments were estimated as described [56]. The PIN184 population has been genotyped among others with the nuclear marker CosA, two allele specific markers derived from the *R3a* and *R3b* late blight resistance genes and SNPs at position 691 and 692 of the *StAOS2* gene, which were associated with field resistance to late blight [56]. Tuber reducing sugar content (RSC) before and after cold storage has been determined in the SUGAR40 panel [53]. Note that although plant maturity (PM) has always been scored by a 1–9 scale, in the BRUISE population PM was scored from very early (score 1) to very late (score 9), while in the GBC and PIN184 populations PM was scored from very late (score 1) to very early (score 9). Where phenotypic data are based on trials in several environments (BRUISE, CHIPS-ALL, PIN184), the adjusted means were used in the present study.

### Determination of cytoplasm types

Genomic DNA was extracted from 10 to 30 mg of freeze-dried leaf tissue from each EURO-CUL genotype using MagAttract 96 DNA Plant kit (Qiagen, Hilden, Germany) and the supplier’s protocol. Cytoplasmic markers T, S, SAC, A and D for cytoplasm type determination and marker detection procedures were performed as described in Hosaka and Sanetomo [32] with the following modifications. The PCR reaction was performed in 5 μl total volume, consisting of 1 μl template DNA (approximately 20 ng/μl), 0.2 mM dNTPs, 2 mM MgCl_2_, 1 U of *Taq* DNA polymerase (PEQ LAB), and 0.5 μl each of 10× forward primer mix and 10× reverse primer mix (6 μM for T, S, and SAC markers and 10 μM for A and D markers). PCR thermal conditions were as follows: 3 min at 94 °C, followed by 35 cycles of 30 s at 94 °C, 30 s at 60 °C, and 1 min and 45 s at 72 °C, and terminated with one cycle of 7 min at 72 °C. After the PCR reaction, the samples were mixed with 5 μl digestion mix including 1 μl 10× FastDigest buffer (Fermentas) and 5 U FastDigest *Bam*HI (Fermentas). Restriction digestions were performed at 37 °C in the thermal cycler for at least 10 min. After *Bam*HI digestion, samples with 10 μl volume were mixed with 3 μl Orange dye (Sigma) and loaded on a 3 % agarose gel in 1× TBE buffer (89 mM Tris–borate, 89 mM boric acid, and 2 mM EDTA). mtDNA types α, β, and γ were distinguished using the ALM_4/ALM_5 primers as described in [30]. 0.5 μl of 10× primer mix in the PCR reaction were replaced by 0.5 μl each of 10 μM ALM_4 and ALM_5 primers. According to Lössl *et al.* [30], presence of a single 2.4 kb band or a single 1.6 kb band corresponds to α-type or β-type mtDNA, respectively, while band absence indicates γ-type mtDNA.

### Statistic analysis

Effects of different cytoplasm types on parametric agronomic traits (TY, TSC, TSY, SCB, rAUDPC and MCR) were analyzed by a one-way analysis of variance (one-way ANOVA) test. If significant differences were found at the 5 % level, cytoplasm types were compared using Tukey’s test. Nonparametric traits showing the difference of the group variances by Bartlett test (PM, TS, CQA, CQS, PM, RLBE, RLBT, RSC and BI) were analyzed using Welch’s test. If significant differences were found at 5 % level, each pair of cytoplasm types was compared by a Kruskal-Wallis test. Note that not all genotypes determined for cytoplasm types had phenotypic data, so that the number of genotypes (n) in the populations differed between traits (Table 3).

Nuclear markers scored in the GBC and PIN184 populations were analyzed for correlation with the presence or absence of the marker for D-type cytoplasm (Table 4). For the SNP marker StAOS2_SNP691/692, a combination of adenine at position 691 and cytosine at position 692 was regarded as the *StAOS2_A*_*691*_*C*_*692*_ haplotype. Allele frequency was calculated by totalizing allele dosage of *StAOS2_A*_*691*_*C*_*692*_ over all genotypes in a group and then dividing by the total number of chromosomes (=number of genotypes in the group × 4). The allele frequencies of different cytoplasm types were compared by a Welch’s analysis. Each pair was compared using Kruskal-Wallis test (Table 5). Combined effects of each nuclear marker and the D-marker on late blight resistance and plant maturity were analyzed. Four marker classes +/+, +/−, −/+, and −/− were tested by Welch’s test for significant differences, where ‘plus’ indicates the presence and ‘minus’ the absence of the marker, irrespective of allele dosage. If significant differences were found among marker classes, two-way ANOVA with the nuclear marker and the D-type cytoplasm as different factors was performed. All statistical analyses were conducted using JMP software (SAS Institute, Inc.).

### Availability of supporting data

The data sets supporting the results of this article are included within the article and its additional files, Additional file 1 and Additional file 2.

## Additional files

Additional file 1: Table S1. The cytoplasm types of all European varieties and breeding clones. Sheet 1 shows the cytoplasm types of European varieties. Sheet 2 shows the cytoplasm types of European breeding clones. Additional file 2: Table S2. A list of duplicated varieties with different cytoplasm types. Sixteen varieties duplicated in at least two populations had different cytoplasm types.

## Abbreviations

PVY: *Potato virus Y*
PVX: *Potato virus X*
PCR: Polymerase chain reaction
QTL: Quantitative trait locus
SNPs: Single nucleotide polymorphisms
*StAOS2*: *Allene Oxide Synthase 2*
mtDNA: Mitochondrial DNA
BI: Bruising index
SCB: Starch corrected bruising
PM: Plant maturity
TS: Tuber shape
TSC: Tuber starch content
TY: Tuber yield
CQA: Chip quality after harvest
CQS: Chip quality after 3 months storage at 4 °C
RSC: Tuber reducing sugar content
RLBF: Foliage resistance to late blight
RLBT: Tuber resistance to late blight
MCR: Maturity corrected resistance
rAUDPC: Relative area under disease progress curve
CIP: Centro International de la Papa (International Potato Center)
