# Supplementary material for: Cytoplasmic genome types of European potatoes and their effects on complex agronomic traits
Source: BMC Plant Biol. 2015 Jun 26;15:162. doi: 10.1186/s12870-015-0545-y (PMC4480903; doi:10.1186/s12870-015-0545-y)
Supplement: Additional file 2: Table S2. — A list of duplicated varieties with different cytoplasm types. Sixteen varieties duplicated in at least two populations had different cytoplasm types. [file 12870_2015_545_MOESM2_ESM.docx]

**Table S2:** A list of duplicated varieties with different cytoplasm types.

| Varieties | Cytoplasm  type |  | Population |  | Cytoplasm  type |  | Population |
| --- | --- | --- | --- | --- | --- | --- | --- |
| Alwara | W/ γ |  | EURO-CUL, BRUISE |  | W/ β |  | GBC |
| Atlanta | T |  | EURO-CUL |  | D |  | GBC |
| Bamberger Hörnchen | A |  | EURO-CUL |  | D |  | GBC |
| Barbara | W/ γ |  | EURO-CUL |  | D |  | GBC |
| Certa | T |  | EURO-CUL |  | W/ γ |  | GBC |
| Deodara | W/ γ |  | EURO-CUL |  | T |  | GBC |
| Diana | D |  | CHIPS-ALL |  | T |  | GBC |
| Heideniere | T |  | EURO-CUL |  | W/ γ |  | GBC |
| Heidrun | W/ γ |  | EURO-CUL |  | W/ β |  | GBC |
| Karolin | D |  | EURO-CUL |  | W/ γ |  | GBC |
| Nora | D |  | EURO-CUL |  | T |  | GBC, BRUISE |
| Pirola | D |  | EURO-CUL |  | T |  | GBC |
| Rebecca | W/ γ |  | EURO-CUL |  | T |  | GBC |
| Saphir | T |  | EURO-CUL |  | D |  | GBC |
| Toccata | W/ γ |  | BRUISE |  | T |  | GBC, EURO-CUL |
| Ute | W/ γ |  | EURO-CUL |  | W/ β |  | GBC |
